# Percutaneous Mechanical Atherectomy Plus Thrombectomy Using the Rotarex®S Device Followed by a Drug-Coated Balloon for the Treatment of Femoropopliteal Artery In-stent Restenosis: A Prospective Single-Center, Single-Arm Efficacy Trial (PERMIT-ISR Trial)

**DOI:** 10.3389/fsurg.2021.671849

**Published:** 2021-09-14

**Authors:** Ming-Yuan Liu, Wenrui Li, Xiaobo Guo, Zhiwen Zhang, Bin Liu, Hongzhi Yu, Zhe Zhang, Xueming Chen, Hai Feng

**Affiliations:** ^1^Department of Vascular Surgery, Beijing Friendship Hospital, Capital Medical University, Beijing, China; ^2^Beijing Center of Vascular Surgery, Beijing, China

**Keywords:** peripheral arterial disease (PAD), femoropopliteal artery disease (FPA), in-stent restenosis (ISR), percutaneous mechanical atherectomy plus thrombectomy (PMAT), drug-coated balloon (DCB)

## Abstract

**Background:** Studies investigating debulking devices with drug-coated balloons (DCBs) in the treatment of femoropopliteal (FP) artery in-stent restenosis (ISR) are limited. We aimed to evaluate the safety and midterm outcome of percutaneous mechanical atherectomy plus thrombectomy (MATH) using the Rotarex®S (Straub Medical, Wangs, Switzerland) catheter followed by a DCB in the treatment of FP-ISR.

**Methods:** This study was a single-center single-arm trial. Patients with symptomatic (Rutherford category 2–5) *de novo* restenosis lesions of FP-ISR were treated with MATH and subsequent DCB. From June 2016 to May 2018, 59 patients with FP-ISR were enrolled. The primary endpoint was target lesion revascularization (TLR) and changes in the Rutherford category of the target limb at 12 months. Secondary endpoints included primary and secondary patency at 12 months, technical success rate, major adverse events, and ankle-brachial index (ABI). Risk factors for TLR were analyzed using Cox proportional hazard model.

**Results:** The average follow-up time was 33 ± 8 months. The rate of technical success was 88.1% (52/59). Nine patients received bailout stenting. The rate of freedom from TLR was 84.7% (50/59) at 1 year, the Rutherford category changed at 12 months were significantly improved from baseline (*p* < 0.01). The primary patency rates and the secondary patency at the 12-month follow-ups were 82.5 and 92.5%, respectively. The ABI changed at 12 months were significantly improved from baseline (*p* < 0.01). Global limb anatomic staging system (GLASS) classification III [hazard ratio (HR) 18.44, 95% CI (1.57–215.99), *p* = 0.020] and postoperative Rutherford classification ≥4 [HR 8.28, 95% CI (1.85–37.06), *p* = 0.006] were identified as independent predictors of TLR.

**Conclusion:** Our preliminary data suggested that MATH using a Rotarex®S catheter combined with DCB angioplasty is a safe, minimally invasive, and effective treatment for FP-ISR with favorable, immediate, and midterm outcomes.

**Clinical Trial Registration:**http://www.chictr.org.cn, identifier [ChiCTR2000041380].

## Introduction

The estimated prevalence of peripheral artery disease (PAD) is up to 200 million worldwide, and self-expanding stents are increasingly used for the treatment of symptomatic femoropopliteal (FP) arterial occlusive disease (1). Despite these clear benefits for stent implantation, in-stent restenosis (ISR) remains a common problem, with an incidence of up to 37% for lesion lengths <150 mm and 60% for longer lesions (2). Many patients with FP-ISR can present with critical limb ischemia (CLI), showing thrombus-containing occlusive lesions triggered by underlying atherosclerotic disease, which has been proven to greatly influence the patency after the endovascular treatment of the lower extremities with a high risk of amputation and potentially severe life-threatening complications.

Recently, several studies have introduced the modality of mechanical debulking (rotational or directional) (3, 4) or drug-coated balloon (DCB) angioplasty (5, 6) for the treatment of FP-ISR and have shown promising outcomes. Despite a lack of clear therapeutic recommendations, the debulking process of the ISR may potentially improve the therapeutic effects of DCBs by reducing the thickness of thrombi and hyperplastic tissue and improving drug delivery to the intima. However, only a few studies (3, 7, 8) with a limited number of cases have reported the results of debulking devices with DCBs for FP-ISR, and their findings are inconclusive. The authors, therefore, aimed to ascertain the safety and midterm outcomes of Rotarex®S rotational atherectomy plus DCB for patients with FP-ISR.

## Methods

### Study Design

Percutaneous mechanical atherectomy plus thrombectomy (MATH) using the Rotarex®S device combined with a DCB for the treatment of FP artery ISR (PERMIT-ISR trial, Registry No. ChiCTR2000041380) is a single-center single-arm pilot trial, which was conducted from June 2016 to May 2018. In this study, all the protocols for using human data were in accordance with the guidelines of the Declaration of Helsinki. Informed consent was obtained from all the patients, and the study protocols were approved by the Institutional Ethical Review Board (No. 2020-P2-073-02). This study was registered in the Chinese Clinical Trial Registry.

### Enrollment

**Figure 1:**
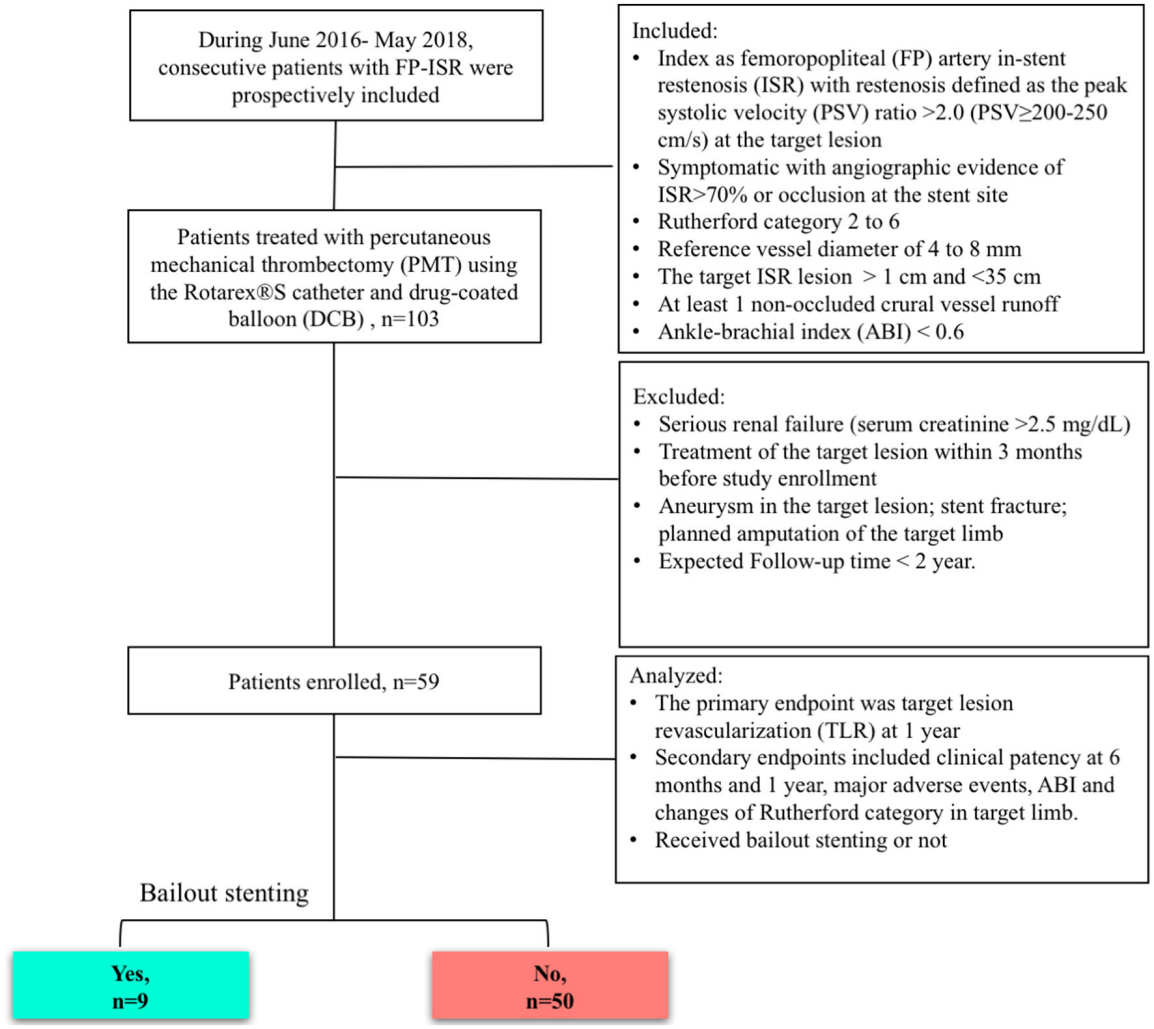
Study diagram. ISR, in-stent restenosis; FP, femoropopliteal; DCB, drug-coated balloon; MATH, percutaneous mechanical atherectomy plus thrombectomy; PSV, peak systolic velocity.

Patients from our center were prospectively enrolled. Operators were trained in adequate use of the devices and had to submit 20 cases for review before they were allowed to participate in the study. The consort flow was shown in Figure 1. The inclusion criteria were FP artery ISR defined as a peak systolic velocity (PSV) ratio > 2.0 (PSV ≥ 200–250 cm/s) at the target lesion (9); angiographic evidence of significant ISR > 70% by visual assessment within the stent; Rutherford category 2 to 6; reference vessel diameter of 4–8 mm; target ISR lesion >1 and <35 cm; at least 1 non-occluded crural vessel runoff; and ankle–brachial index (ABI) <0.6. The exclusion criteria were serious renal failure (serum creatinine > 2.5 mg/dL), treatment of the target lesion within 3 months before study enrollment, aneurysm in the target lesion, stent fracture, planned amputation of the target limb, and expected follow-up time <2 years.

There were 59 symptomatic patients enrolled (39 men and 20 women) with ISR of a previously implanted stent who underwent MATH (Rotarex®) and DCB angioplasty treatment. The mean age was 71.0 ± 11.2 years (median, 69 years), ranging from 55 to 86 years. Risk factors and comorbidities of patients, such as high blood pressure, diabetes, smoking, hyperlipoproteinemia, heart disease, and chronic renal failure, were collected (Table 1). A total of 55 cases (93.2%) were using antiplatelet treatment aspirin 100 mg qd before presenting to department. Limb ischemia was categorized according to the Rutherford classification (4). Patients presented with limb ischemia in Rutherford category 2–3 in 11 cases (18.6%) and Rutherford category 4–6 in 48 cases (81.4%). According to the reported standard (10), 83.0% of this cohort were patients with CLI.

**Table 1:** Baseline clinical characteristics (*N* = 59).

**Variable**	**Value**
Age, y	71.0 ± 11.2
Gender (M/F)	39/20
Comorbidities	
Smoker, *n* (%)	26 (44.1)
Diabetes mellitus, *n* (%)	27 (45.8)
Hypertension, *n* (%)	40 (67.8)
Hypercholesterolemia, *n* (%)	35 (59.3)
Coronary artery disease, *n* (%)	17 (28.8)
Cerebrovascular disease, *n* (%)	6 (10.2)
Renal impairment, *n* (%)	8 (13.6)
Dialysis, *n* (%)	2 (3.4)
Obesity, *n* (%)	11 (18.6)
Cardiopathy, *n* (%)	13 (22.0)
Patency of stenting (month)	13.2 ± 9.1
Number of stents	1.9 ± 0.6
Size of stents (mm)	5.1 ± 0.3
Antiplatelet treatment before restenosis, *n* (%)	
Aspirin 100mg per day	55 (93.2)
Stop medication due to bleeding	4 (6.8)
Onset of ischemia (day)	46.4 ± 17.3
Rutherford classification, *n* (%)	
0–1	0
2–3	11 (18.6)
4–6	48 (81.4)
TASC classification	
A	4 (6.8)
B	8 (13.6)
C	16 (27.1)
D	31 (52.5)
In-stent lesion length (cm)	18.4 ± 7.8
Mehran's classification of ISRs, *n* (%)	
I	4 (6.8%)
II	3 (5.1)
III	10 (16.9)
IV	42 (71.2)
GLASS classification for CLI	
I	12 (20.3)
II	25 (42.3)
III	22 (37.4)
Critical limb ischemia	49 (83.1)
Number of runoff vessels, *n* (%)	
1	34 (57.6)
2	17 (28.8)
3	8 (13.6)
Thrombus detected by CDU, *n* (%)	40 (67.8)
Target vessel diameter (mm)	4.88 ± 7.86
ABI	0.38 ± 0.10

*CDU, color duplex ultrasonography; TLR, target lesion revascularization; ISR, in-stent restenosis; TASC, TransAtlantic Inter-Society Consensus; GLASS, Global Limb Anatomic Staging System; ABI, ankle/brachial index*.

### Study Devices and Procedural Characteristics

Patients were classified according to Mehran's classification (11) and GLASS classification (10) of their occlusion (Table 1). The MATH device used was the Rotarex®S System (Straub Medical, Wangs, Switzerland), with 6- or 8-French (Fr) sheath compatible devices, depending upon the vessel diameter to be treated. The length of the lesions was visually estimated on digital subtraction angiography. The DCBs used were Orchid® paclitaxel-coated balloons (Orchid, AcoTec, Beijing, China) with diameters of 4–6 mm and lengths of 120–300 mm. Bailout stents included Smart Flex (Cordis, USA), Pulsar-18 (Biotronik, Woermannkehre, Berlin, Germany), and VIABAHN (W. L. Gore & Associates, USA).

**Figure 2:**
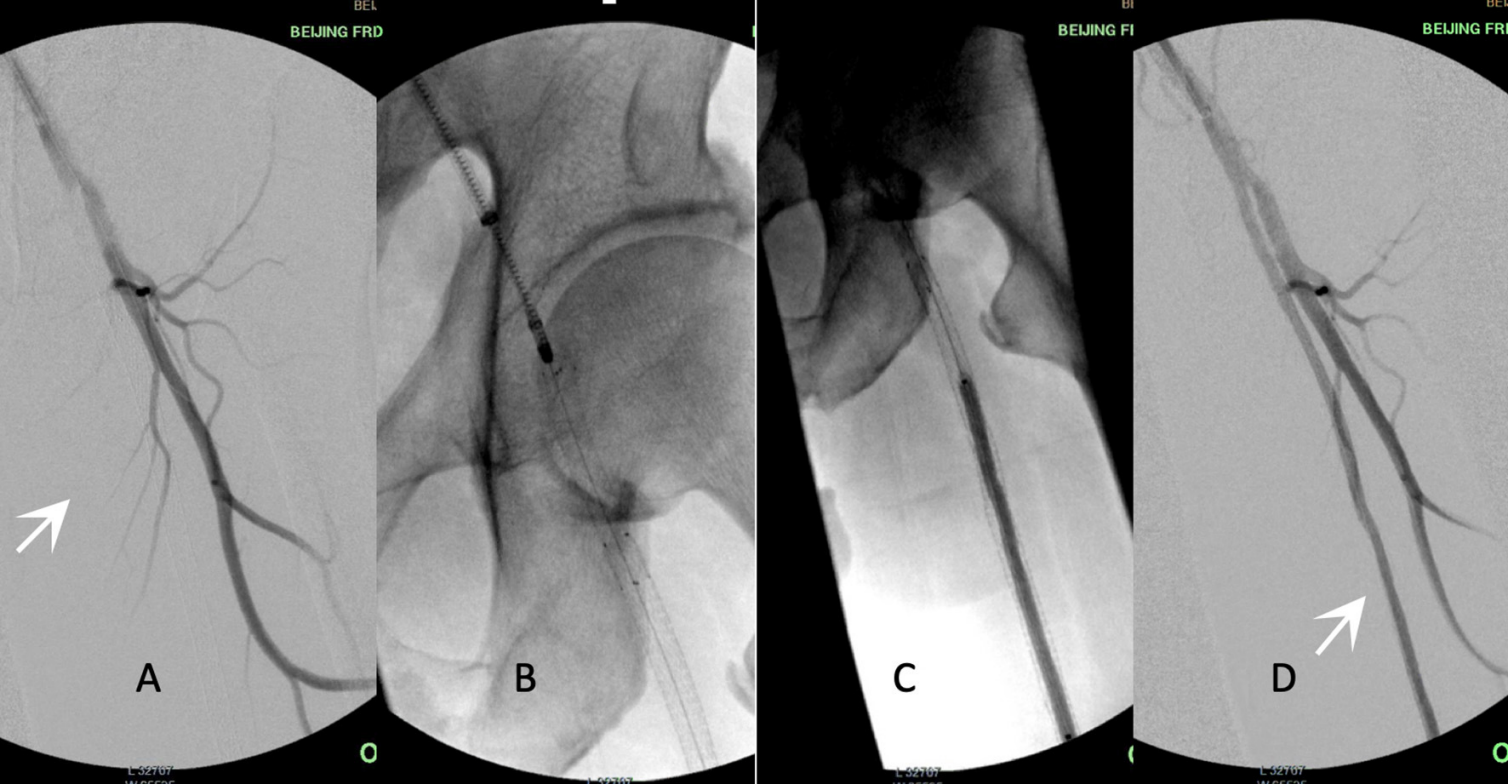
Procedure illustration. Acute in-stent restenosis (ISR)/occlusion of femoropopliteal (FP) lesions treated by percutaneous mechanical atherectomy plus thrombectomy (MATH) using Rotarex®S combined with a drug-coated balloon (DCB). **(A)** White arrow shows stent occlusion from the superficial femoral artery to the popliteal artery. **(B)** The 8F rotational Rotarex® S catheter was advanced into the proximal site of the stent. **(C)** DCB angioplasty of the FP-ISR after the debulking process. **(D)** The final result of angiography after adjunctive MATH and DCB. White arrow shows the patent FP artery. ISR, in-stent restenosis; FP, femoropopliteal; DCB, drug-coated balloon; MATH, percutaneous mechanical atherectomy plus thrombectomy.

The approaches performed during the interventions included common femoral ipsilateral, common femoral contralateral, or brachial access. The Rotarex®S device was inserted over a 0.018-inch guidewire until reaching a few centimeters proximal to the lesion. Several passes were necessary to eliminate the thrombus. Transluminal angioplasty with uncoated balloons was predilated (2 min) with 0.5–1 mm smaller than the reference vessel (visually estimated) before the DCB process. DCBs were inflated only once for 3 min at the recommended atm (Figure 2). A bailout stent was implanted if the residual stenosis diameter was >50% under intraoperative angiography.

The mean runtime was 10.4 ± 5.6 min with the Rotarex®S device (Table 2). An appropriate closure device was used to obtain hemostasis in the cases of complications. Postinterventional medication included anticoagulation therapy as a curative or preventive measure. After the procedure, single or dual antiplatelet therapy (aspirin 100 mg/day and clopidogrel 75 mg/day) was recommended for at least 12 weeks to avoid thrombotic events.

**Table 2:** Procedural characteristics and clinical outcome at 12 months.

**Variable**	**Value**
Total vascular access sites, *n* (%)	
Crossover	56 (94.9)
Antegrade	3 (5.1)
Rotarex system selection, *n* (%)	
8F	48 (81.4)
6F	11 (18.6)
Duration of MATH (minute)	10.4 ± 5.6
Blood loss (ml)	86.6 ± 34.8
Blood transfusion case, *n* (%)	0
Recanalization techniques, *n* (%)	
MATH-DCB	50 (84.7)
MATH-DCB+bailout stenting	9 (15.3)
TLR at the 12 months	9 (15.3)
Major complications, *n* (%)	
Perforation	0
Embolization	3 (5.1)
Dissection	2 (3.4)
Thrombosis	0
Bleeding	0
False aneurysm	0
Infection	0
Major adverse events, *n* (%)	
Death	2 (3.4)
MI	2 (3.4)
Major amputation	1 (1.7)
Renal failure	0
Stroke	0
Rutherford classification, *n* (%)	
0–1	17 (28.8)
2–3	35 (59.3)
4–6	7 (11.9)
ABI postoperative	0.83 ± 0.33 0.71 ± 0.16***
ABI at the 12 months	

MATH, percutaneous mechanical atherectomy plus thrombectomy; DCB, drug-coated balloon; MI, myocardial infarction; TLR, target lesion revascularization; ABI, ankle/brachial index (compared to preoperative ABI); ***P < 0.001.

### Follow-Up and Endpoints

The patients were routinely scheduled to return for ambulatory follow-up visits examined by the color duplex ultrasonography (CDU) at 30 days after hospital discharge and then at 3, 6, 12, 18, and 24 months. Stent integrity was investigated with straight-leg flat plate X-rays or fluoroscopy at 6 and 12 months. The primary endpoint was target lesion revascularization (TLR) and changes in the Rutherford category of the target limb at 12 months. Secondary endpoints included primary patency (freedom from clinical driven TLR and restenosis > 50%) and secondary patency (patency after the TLR) at 6 and 12 months, major adverse events (MAEs) and changes of ABI, as well as whether the patients received bailout stenting or not. Other outcome measures were technical success, limb salvage, and survival. Technical success was defined as successful completion of the procedure and recanalization of the entire occlusion length with ≤ 30% diameter residual stenosis. Clinical success for patients with claudication (Rutherford II-III) was defined as clinical improvement in at least one clinical category; for the patients with CLI (Rutherford IV–VI), clinical success was defined as the resolution of ischemic rest pain and the healing of ischemic ulcers.

The MAEs were defined as death, myocardial infarction (MI), stroke, renal failure, and major amputation, occurring between the perioperative and follow-up periods. The ABI of each patient was documented perioperatively and examined 1 day after the procedure. TLR was defined as any repeat percutaneous intervention of the target lesion or bypass surgery of the target vessel performed for restenosis or other complication of the target lesion.

### Statistical Methods

Continuous variables are summarized as the mean ± SD. Categorical variables are reported as counts and percentages. The Cochran–Armitage test was used to calculate *p*-values for a trend in the Rutherford category change. Kaplan–Meier analyses assessed freedom from TLR, primary patency, and secondary patency over time. A paired *t*-test was used to compare the preoperative ABI to the postoperative and follow-up values. Cox proportional hazard regression analyses were used to determine potential risk factors for TLR after MATH and DCB. Statistical analysis was performed using SAS (version 9.4; SAS Institute Inc., Cary, NC, USA) and R (R Foundation for Statistical Computing, Vienna, Austria; http://www.r-project.org). *P* < 0.05 was considered statistically significant.

## Results

### Baseline Characteristics

During the study period, 59 patients with FP-ISR underwent a combination of MATH (Rotarex®) and DCB angioplasty. The clinical features of the patients are presented in Table 1. There were 59 limbs (36 right and 23 left) with lesions that had received stent implantation, and the patency of stenting ranged from 4 to 62 months with an average of 13.2 ± 9.1 months. More than one stent was placed in over 85% of patients with an average size of 5.1 ± 0.3 mm. Over 80% of our patients had a Rutherford score of IV-VI at admission. Ischemia occurred in a range of 2–90 days following the stenting procedure, with an average onset at 46.4 ± 17.3 days. Computed tomography angiography (CTA) showed an average lesion length of 18.4 ± 7.8 cm with a range of 8.6–32.4 cm. The majority of patients presented with an in-stent occlusion (*n* = 42; 71.2%). The preoperative ABI range was 0.24–0.53, with an average of 0.38 ± 0.10. The CDU revealed that 40 limbs (67.8%) had a thrombus in the stent or at the distal end of the stent.

### Early Outcome

The early outcomes are presented in Table 2. MATH was successfully completed in all the patients (59 cases). The median runtime of Rotarex®S lasted 10.4 ± 5.6 min. Technical success ( ≤ 30% residual stenosis following protocol treatment, before adjunctive treatments) was achieved in 88.1% (52/59). The clinical success rate was 93.2% (55/59). Nine patients received bailout stenting after the MATH-DCB procedures, among which four cases were reimplanted with a stent into the original stenotic stent, two for vascular perforation, and three cases were reimplanted with a stent into the restenosis spots at the distal end of the original stent. None of the cases required blood transfusion or intraoperative catheter direct thrombolysis. Distal embolism occurred in three cases (5.1%) during the intervention and was extracted using an endovascular technique with a 5F guidewire catheter. The operative details and overall angiographic data are outlined in Table 2. The average ABI increased from 0.38 ± 0.10 before the operation to 0.83 ± 0.33 1 day after the procedure (*P* = 0.001).

### Follow-Up

**Figure 3:**
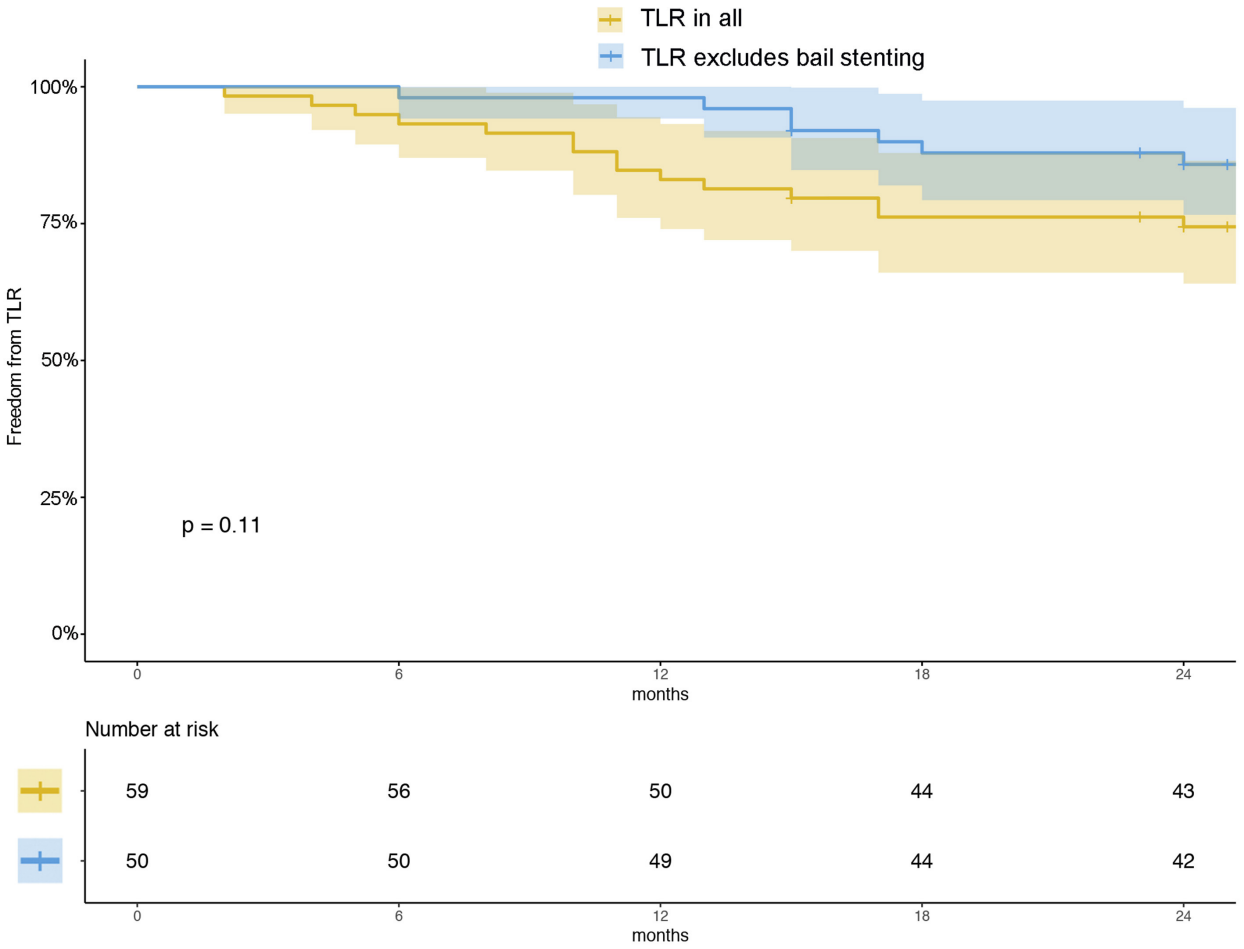
Kaplan–Meier estimates of freedom from TLR (yellow) and freedom from TLR excluding patients who received bailout stents (blue) at 12 months. The curve shows that the rate of freedom from TLR for all patients was 84.7% and that of freedom from TLR excluding the patients who received bailout stents was 96% at 12 months. The shaded area represents the 95% CI.

**Figure 4:**
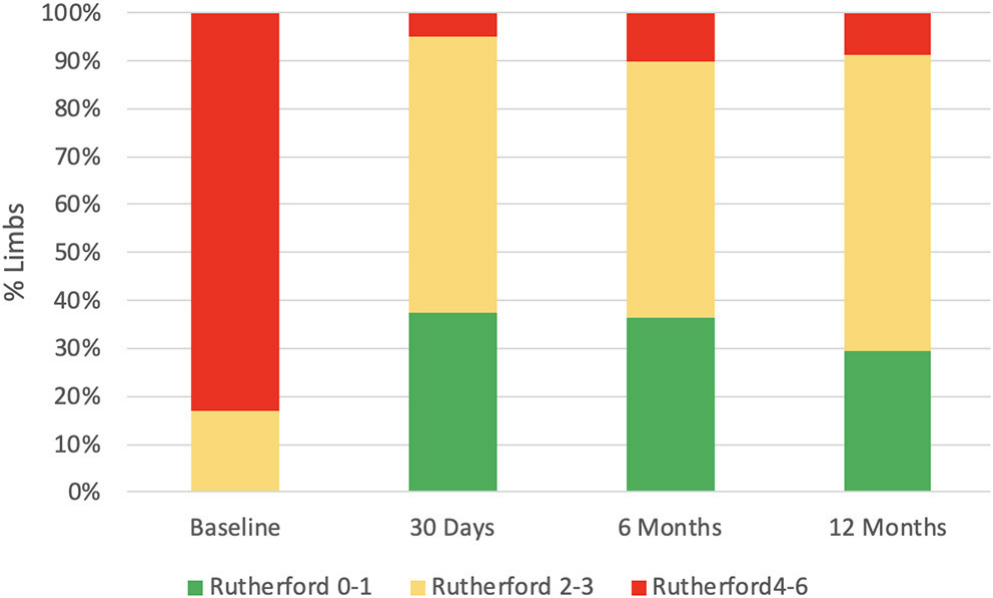
The Rutherford category significantly improved from baseline (*p* < 0.01).

**Figure 5:**
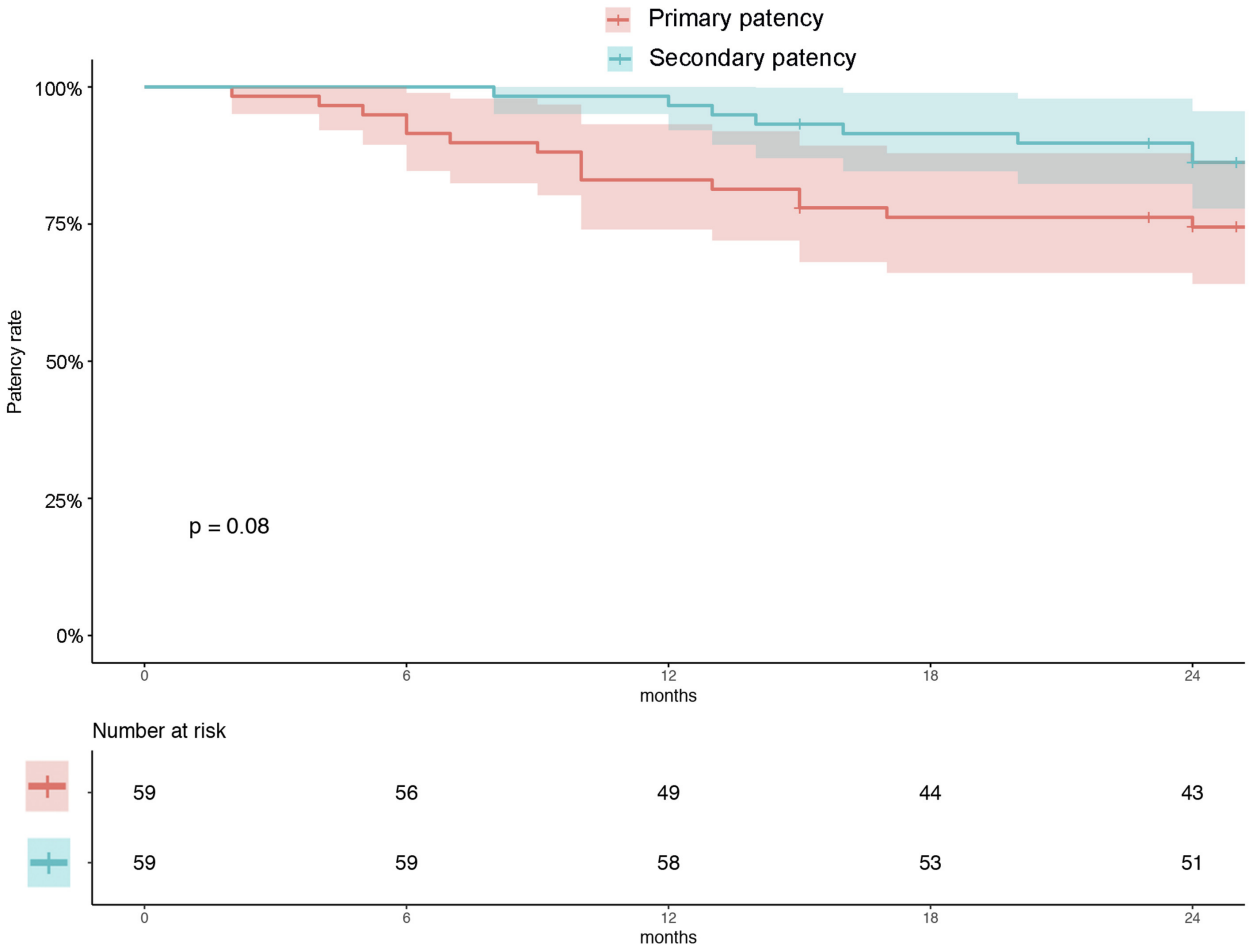
Kaplan–Meier estimates of primary patency (red) and secondary patency (green) rates at 12 months. The curve shows that the primary and secondary patency rates were 82.5 and 92.5%, respectively, at 12 months. Primary patency was defined as patency, freedom from clinical driven TLR, and restenosis >50%. Secondary patency was defined as patency after TLR. The shaded area represents the 95% CI. TLR, target lesion revascularization.

There were 54 (93.2%) patients who received follow-up for at least 24 months after the procedures. The average follow-up time was 33 ± 8 months, and ranged from 24 to 49 months. Kaplan–Meier estimates of the rate of freedom from TLR were 84.7% (50/59) at 1 year. The rate of freedom from TLR excluding patients who received bailout stents (9) at 1 year was 96% (48/50). There was no significant difference between the two groups (0.11, Figure 3). The Rutherford category at the 12-month follow-up was significantly improved from baseline (*p* < 0.01, Figure 4). The primary patency at 6 and 12 months postoperatively was 91.4 and 82.5%, and the secondary patency rates were 100 and 92.5%, respectively (*P* = 0.08, Figure 5). During the follow-up, ISR occurred in 17 cases (28.8%), at an average of 246 ± 82 days after the procedure. Of these 17 patients, 11 patients received MATH (Rotarex®S) combined with DCB angioplasty again, and six patients were treated conservatively with medication therapy only. Limb salvage during the follow-up period was performed in 98.3% (58/59) of patients. Two patients, however, died of acute myocardial infarction. The ABI significantly improved from 0.38 ± 0.10 before the operation to 0.71 ± 0.16 at the 12-month follow-up (*P* < 0.001). The MAEs were two deaths (2/59, 3.4%), two MIs (2/59, 3.4%), and one major amputation (1/59, 1.7%) during the perioperative period. The major complications and MAEs are presented in Table 2. We performed a Cox proportional hazard regression analysis to determine potential risk factors for TLR after MATH with DCB (Table 3). In univariate analyses, length of ISR ≥ 20 cm, GLASS classification III, postoperative Rutherford classification ≥ 4, bailout-stent implantation was significantly associated with increased risk of TLR. However, in a multivariate analysis, only GLASS classification III [HR 18.44, 95% CI (1.57–215.99), *p* = 0.020] and postoperative Rutherford classification ≥ 4 [HR 8.28, 95% CI (1.85–37.06), *p* = 0.006] were identified as significant independent risk factors of TLR after MATH with DCB treatment. However, the Mehran classification of ISR III/IV did not have an independent impact on the risk for TLR.

**Table 3:** Cox proportional hazard regression analysis for TLR.

**Variables**	**Univariate analysis**		**Multivariate analysis**	
	**HR (95% CI)**	***P*-value**	**HR (95% CI)**	***P*-value**
Length of ISR ≥20 cm	9.61 (2.38–38.84)	0.001		
Hypercholesterolemia	56.30 (0.26–144.16)	0.143		
Mehran's classification of ISRs III/IV	1.00 (1.00–1.01)	0.237		
GLASS classification III	18.27 (2.27–147.16)	0.006	18.44 (1.57–215.99)	0.020
Postoperative Rutherford classification ≥4	16.04 (4.04–63.72)	<0.001	8.28 (1.85–37.06)	0.006
Bailout-stent implantation	4.66 (1.25–17.40)	0.022		

*TLR, target lesion revascularization; ISR, in-stent restenosis; GLASS, Global Limb Anatomic Staging System; Postoperative Rutherford classification indicated the evaluation result at 12 months after the procedure*.

## Discussion

Acute and subacute life-endangering limb ischemia could be driven by reduced arterial perfusion to a limb due to ISR (12). Despite the refinement of endovascular techniques, the role of endovascular therapy in the treatment of PF-ISR remains intractable. However, this study has shown a favorable outcome of MATH combined with DCB for FP-ISR with the rate of freedom from TLR were 84.7% (50/59) at 1 year.

In-stent restenosis lesions are associated with refractory restenosis and remarkably differ from *de novo* atherosclerotic lesions; restenotic tissue consists primarily of a collagen-rich extracellular matrix with an inner layer of smooth muscle cells (13) that has limited the therapeutic effect of endovascular techniques. Recently, mechanical thrombectomy devices including the MATH that have the advantage of lower invasiveness, lower risk of complications, and shorter treatment duration, are available as an alternative approach (12). A systematic review and meta-analysis suggested that debulking devices combined with DCBs may be a promising way for FP-ISR complex lesions (14).

In-stent restenosis is often a luminal occlusion caused by the formation of thrombi based on intimal hyperplasia of the lumen (15, 16). An adjunctive thrombus is considered as a major limiting factor for treating patients with FP stenosis or occlusion *via* a DCB-alone approach (17). This is an appropriate application of the characteristics of the debulking device of the Rotarex®S. The Rotarex®S system is an effective MATH device and represents a minimally invasive approach for quickly recanalizing thrombus-containing lesions regardless of the age of the thrombus (12). However, atherectomy alone for FP-ISR may be an insufficient choice. The EXCITE ISR trial (18) was the first large, randomized, and prospective study to evaluate the advantages of laser atherectomy with PTA vs. PTA alone. The results showed (18) that at 6 months, compared with PTA, the laser atherectomy method had a higher rate of patency (71.1 vs. 56.4%, *P* = 0.004) and a lower rate of TLR (20.2 vs. 36.3%, *P* = 0.003). However, similar to DCBs, atherectomy did not increase the ABI significantly or improve the ischemic condition (19). Although atherectomy has advantages over other treatments in terms of major amputation and all-cause mortality, a meta-analysis has shown that atherectomy alone seems to be less effective for FP-ISR (20). This is because simple debulking therapy, such as atherectomy, injures the vascular endothelium and intensifies smooth muscle cell proliferation in ISR lesions, which causes a high recurrence rate. Similarly, DCB is a method of balloon angioplasty, and for the severe ISR/occlusion of substantial hyperplastic tissues, its antiproliferative effect cannot be effectively exerted (21). In a meta-analysis of ISR, Hajibandeh et al. suggested that compared with PTA, both atherectomy and DCB improved delivery but yielded crude outcomes (22).

To date, there have been no trials comparing the efficiency and safety of atherectomy with DCBs with DCBs alone. There is a complementarity between atherectomy and DCB therapy. Therefore, in this study, we conducted a single-arm trial of rotational atherectomy with subsequent DCB to evaluate the advantages of both removal of occlusive tissue and anti-proliferative effects. In this study, we used MATH (Rotarex®S catheter) combined with DCB in the treatment of FP-ISR. In our cohort, the TLR at 1 year was 15.3%, the primary patency was 82.5%, and the secondary patency was 92.5%.

Although reports of DCB angioplasty or MATH offer alternative approaches to established ISR treatment (13, 19, 23), clinical evidence of the combination of these two modalities is scarce. DEFINITIVE AR was the first prospective, multicenter study designed to estimate the effect of treating FP-ISR with the combination of the directional atherectomy (DA) and DCB (4). The 1-year primary patency rate was 84.6% for the DA + DCB group in DEFINITIVE AR that was significantly higher than that of the DCB group (4). Loffroy et al. (3) conducted a multicenter retrospective study using a Rotarex®S rotational debulking device (MATH) alone or associated with percutaneous transluminal angioplasty (PTA) and/or DCB angioplasty for the treatment of FP-ISR. At the 12-month follow-up, the primary and secondary patencies were 92.7 and 91.4% (after reintervention), respectively, with a TLR rate of 25 of 128 (19.5%). Our results, in inconsistency with the outcome of the previous reports, have shown the favorable outcome of MATH combined with DCB for FP-ISR.

Perforation consequence of MATH (especially for the long lesions) is considered as an unfavorable situation where bailout stenting is required (5). In this study, nine patients received bailout stenting after MATH + DCB. Kaplan–Meier estimates of the rate of freedom from TLR for all the patients at 1 year was 84.7% (50/59), whereas the rate of freedom from TLR excluding patients who received bailout stents at 1 year was 96% (48/50). However, there was no significant difference between the groups (*P* = 0.11, Figure 3). In the univariate Cox analysis, the bailout stenting seemed to be a risk factor for TLR after the MATH with DCB [HR 4.66, 95% CI (1.25–17.40), Table 3]. However, the trend was corrected in the multivariate analysis (*P* > 0.05). These results demonstrated that TLR after MATH + DCB was not significantly affected by the initial implantation of the bailout stent.

In addition, using the Kaplan–Meier method, we evaluated the primary (82.5%) and secondary patency (92.5%) at 1 year (Figure 4), and no significant difference was found during the follow-up (*P* = 0.08). Despite reintervention for TLR, this result showed acceptable primary patency up to the 1-year follow-up.

Our results have shown that GLASS classification III [HR 18.44, 95% CI (1.57–215.99), *p* = 0.020] and postoperative Rutherford classification ≥ 4 at 12 months [HR 8.28, 95% CI (1.85–37.06), *p* = 0.006] were the independent risk factors for TLR. It suggested that preoperative long-range loss of target arterial path and postoperative rest pain or tissue loss were significant indicators for TLR. The result has demonstrated the improvement of preoperative GLASS classification, and the postoperative Rutherford stage may be important for avoiding the TLR. Data indicated that another advantage of MATH with DCB treatment was that the ABI (*P* < 0.001, Table 2) and Rutherford category at the 12-month follow-up were significantly ameliorated from baseline (*p* < 0.01, Figure 4). In contrast with our results, those of another study showed that the long-term patency rate was not satisfactory for the patients who underwent MATH combined with plain balloon angioplasty (24). The Rutherford classification appeared to persist in 74.1% of the patients over a 12-month period in the treatment of 525 patients with acute and subacute occlusions in native arteries (16). The restenosis rate was higher after MATH with uncoated balloon angioplasty than after DCB (25). Although there was no comparison group, the combination of the Rotarex®S catheter and DCB used in this investigation achieved an acceptable patency rate in comparison with that in other large comparative studies (26, 27).

This study has some limitations. First, it is limited by the relatively small number of patients enrolled and the absence of a control group. Therefore, the comparisons were insufficiently made by our cohort and published data. Selection bias existed in this study because the included patients were not assigned randomly. Patients were excluded because of several criteria. Second, this study determined that the MATH and DCB had a low-favorable outcome, but we did not investigate whether certain subgroups of the patients or lesions profited the most, given the small sample size available. Third, the combination of atherectomy (rotational or directional) with DCB was insufficiently investigated. Several types of DCBs and mechanical thrombectomy devices are currently available in the market. Therefore, large-scale randomized controlled trials are required to further evaluate the efficiency and safety of this combined method with different compositions of devices.

## Conclusion

In patients with ISR of the FP artery, MATH using a Rotarex®S catheter followed by DCB angioplasty is a safe, minimally invasive, and effective treatment with favorable immediate and midterm outcomes. Bailout stenting after the MATH and DCB procedures may be an acceptable remedy for lowering the TLR.

## Data Availability Statement

The raw data supporting the conclusions of this article will be made available by the authors, without undue reservation.

## Ethics Statement

The studies involving human participants were reviewed and approved by Institutional Ethical Review Board of Beijing Friendship Hospital (No. 2020-P2-073-02). The patients/participants provided their written informed consent to participate in this study. Written informed consent was obtained from the individual(s) for the publication of any potentially identifiable images or data included in this article.

## Author Contributions

M-YL, XC, and HF contributed to the conception and study design and obtained funding. M-YL and WL contributed to writing the article. XC, HY, ZheZ, BL, and ZhiZ contributed to the critical revision of the article. M-YL, WL, and XG helped in the data collection, analysis, and interpretation. All authors contributed to the article and approved the submitted version.

## Funding

This work was supported by the Capital Health Research and Development of Special (2020–2–1102), the National Natural Science Foundation of China (No. 82000429), the Beijing Municipal Administration of Hospitals Incubating Program (PX2021002), and the Scientific Research Program of Beijing Education Commission (KM202110025016).

## Conflict of Interest

The authors declare that the research was conducted in the absence of any commercial or financial relationships that could be construed as a potential conflict of interest.

## Publisher's Note

All claims expressed in this article are solely those of the authors and do not necessarily represent those of their affiliated organizations, or those of the publisher, the editors and the reviewers. Any product that may be evaluated in this article, or claim that may be made by its manufacturer, is not guaranteed or endorsed by the publisher.
